# Supplementary material for: Working memory performance is tied to stimulus complexity
Source: Commun Biol. 2023 Nov 3;6:1119. doi: 10.1038/s42003-023-05486-7 (PMC10624839; doi:10.1038/s42003-023-05486-7)
Supplement: Supplementary file 2 — Supplementary Material [file 42003_2023_5486_MOESM2_ESM.docx]

**Supplementary Information**


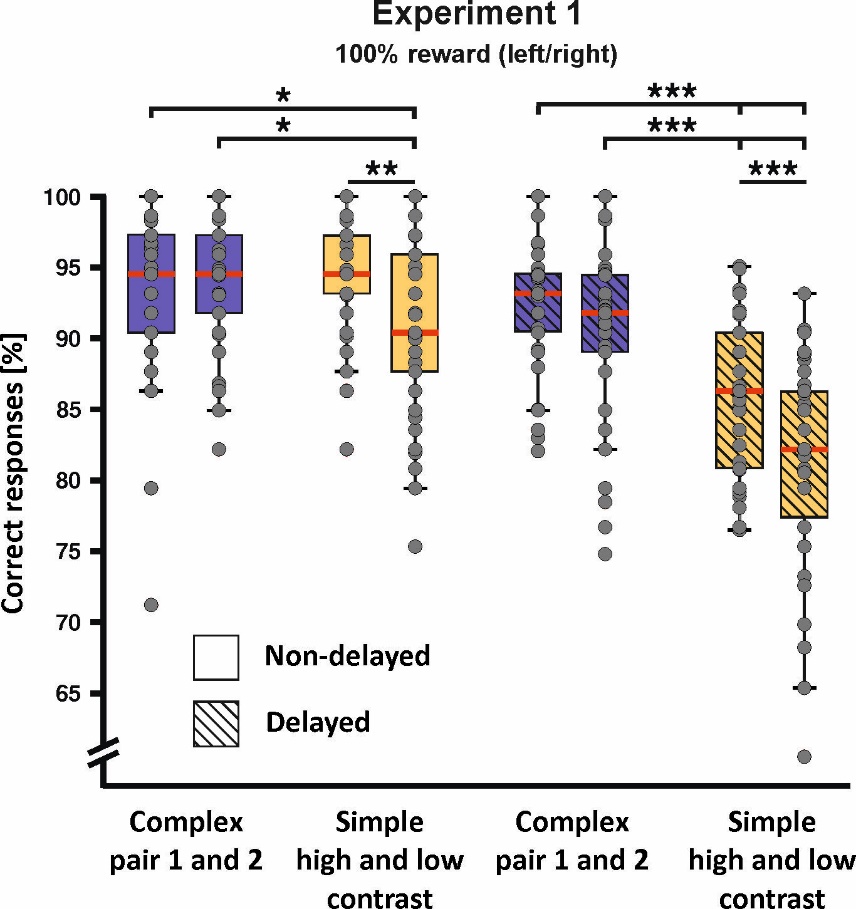


**Supplementary Figure 1. Behavioral performance of experiment 1 for all individual stimulus pairs.**

The introduction of the delay had differential effects on performance across stimulus pairs. Complex pairs 1 and 2 refer to the two different pairs of complex stimuli in our study. *p < 0.05; **p < 0.01; ***p < 0.001. Error bars represent the 95% confidence interval.


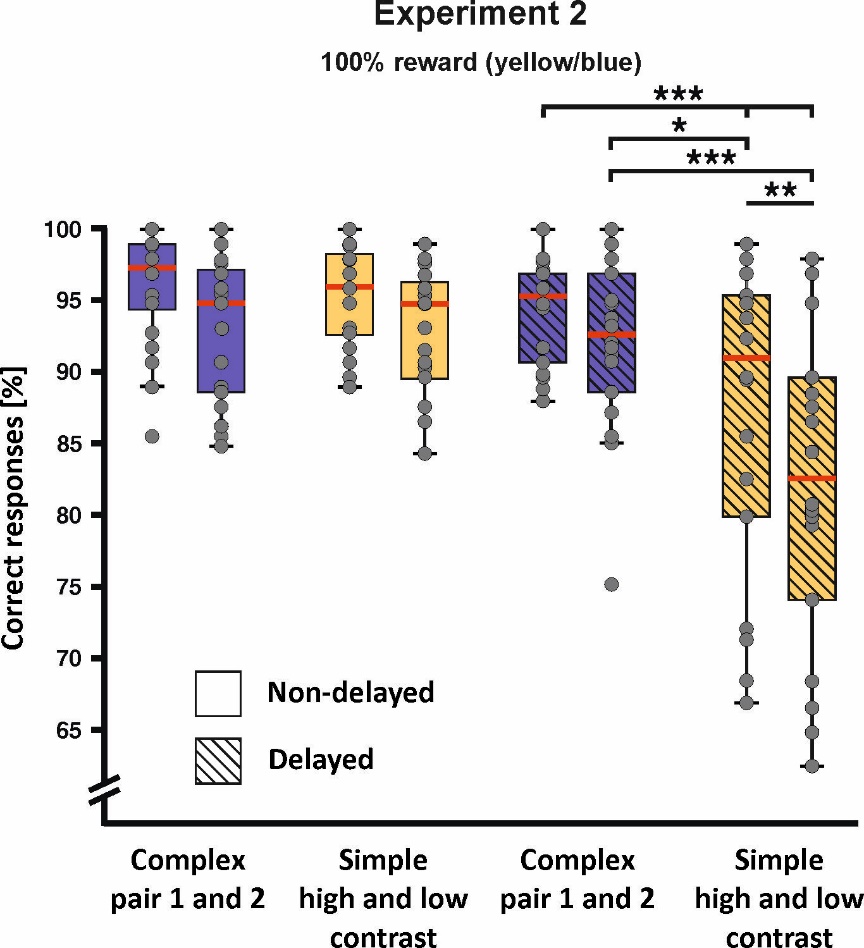


**Supplementary Figure 2. Behavioral performance of experiment 2 for all individual stimulus pairs.**

The introduction of the delay had differential effects on performance across stimulus pairs. Complex pairs 1 and 2 refer to the two different pairs of complex stimuli in our study. *p < 0.05; **p < 0.01; ***p < 0.001. Error bars represent the 95% confidence interval.


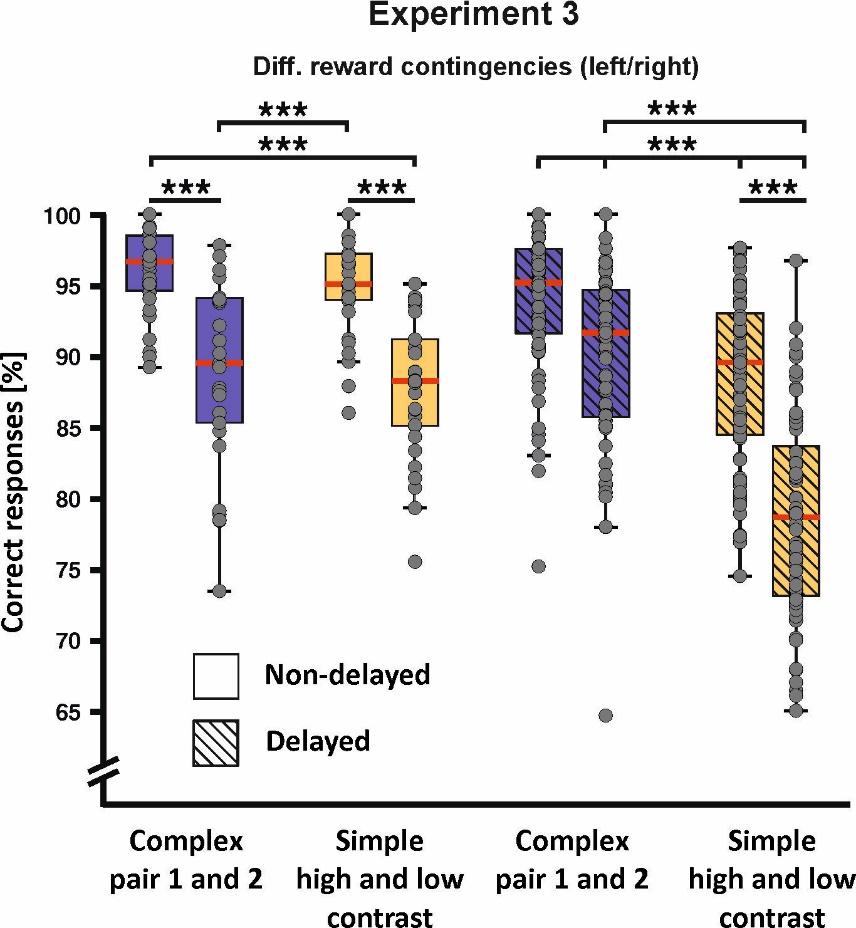


**Supplementary Figure 3. Behavioral performance of experiment 3 for all individual stimulus pairs.**

The introduction of the delay had differential effects on performance across stimulus pairs. Complex pairs 1 and 2 refer to the two different pairs of complex stimuli in our study. ***p < 0.001. Error bars represent the 95% confidence interval.


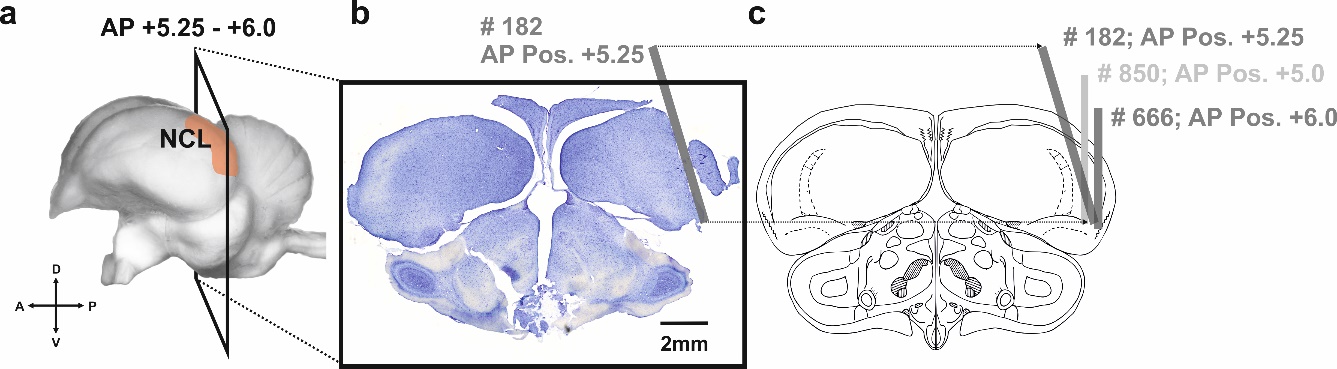


Supplementary Figure 4. Histological analysis of electrode tracks for each individual pigeon subjected in the study.

a Sagittal view of the pigeon brain^1^ including the range of coronal planes of the electrode positions. b Example of the electrode track reconstruction for pigeon # 182 in a Nissl stained brain slice cut at 40µm. c Schematic electrode track reconstruction for all pigeons used in this study collapsed on a coronal section of the pigeon brain. Drawings are based on the pigeon brain atlas by Karten and Hodos^2^. Gray lines indicate the location of the cannula containing the electrodes for each individual pigeon (Pigeon # 182, # 850, and # 666). For all three pigeons, the electrode tracks were located within the borders of the NCL as defined by Herold et al.^3^. A: anterior; AP: anterior-posterior axis; D: dorsal; NCL: nidopallium caudolaterale; P: posterior; V: ventral.

.
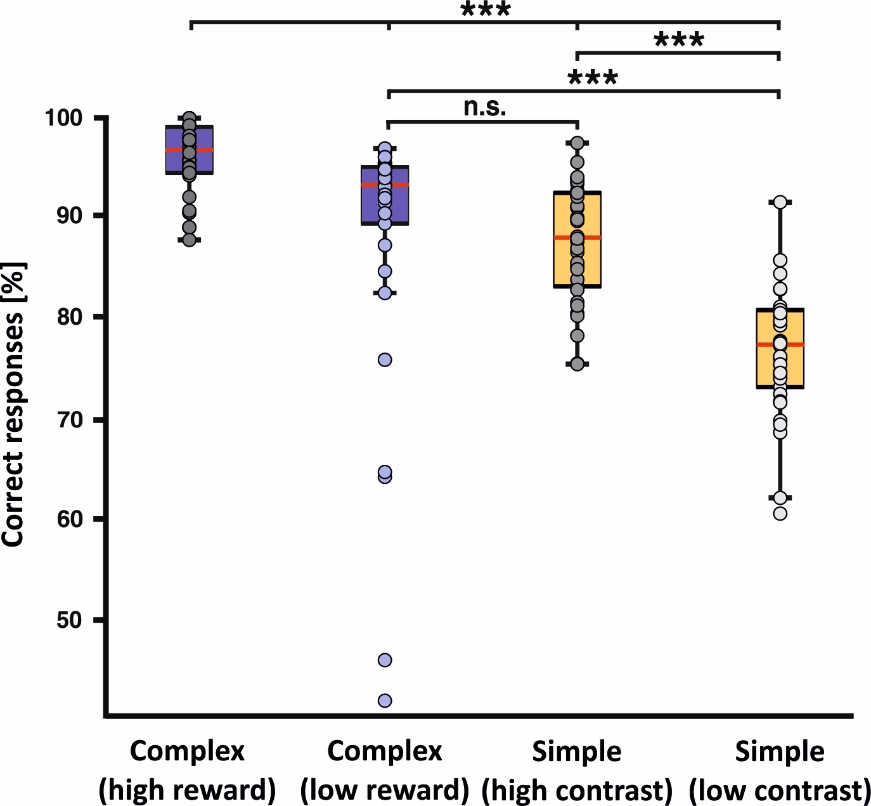


**Supplementary Figure 5. Behavioral performance during the recording sessions (only the delayed condition was run).**

Performance levels echoed the results of experiment 3 in the delayed condition (see Figure 2c). ***p < 0.001. Whiskers represent Q1 − 1.5 * interquartile range (IQR) and Q3 + 1.5 * IQR.


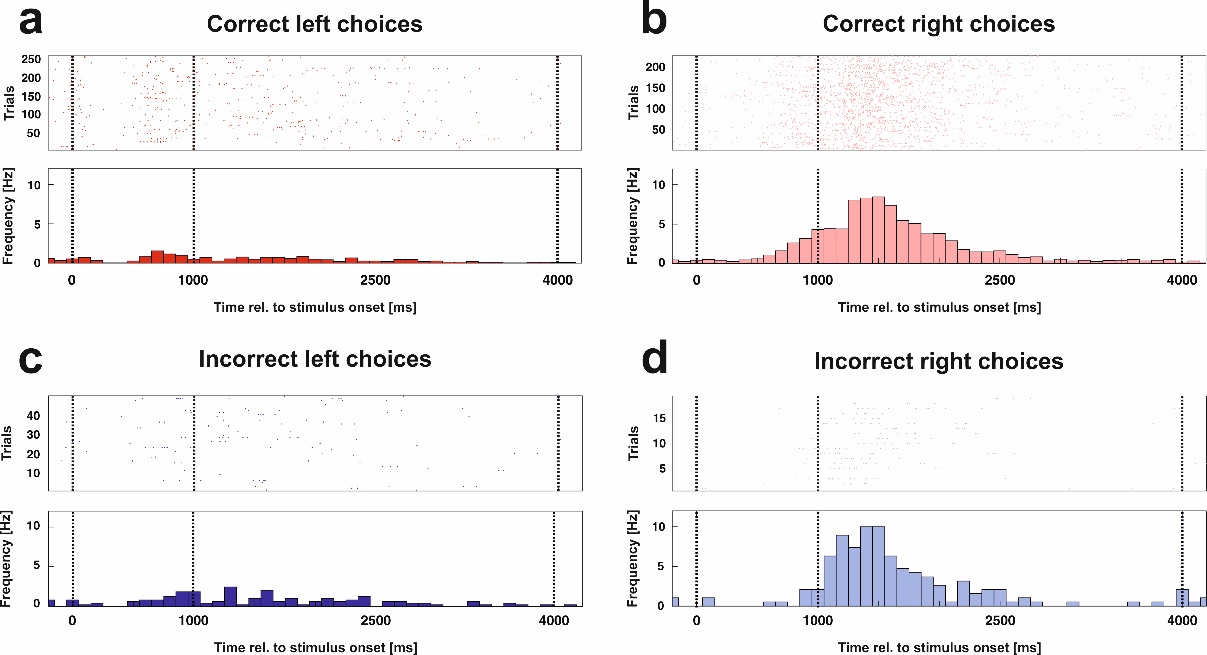


Supplementary Figure 6. Responses of an example neuron for all left and right choices across all experimental stimuli.

a The raster plot and PSTH for correct choices to the left response key are shown. b The corresponding responses to the right response key are depicted. c The raster plot and PSTH for incorrect choices to the left response key are shown. d The corresponding responses to the right response key are depicted. Note that the neuron was tuned to the ultimate decision irrespective of the outcome (correct vs. incorrect).


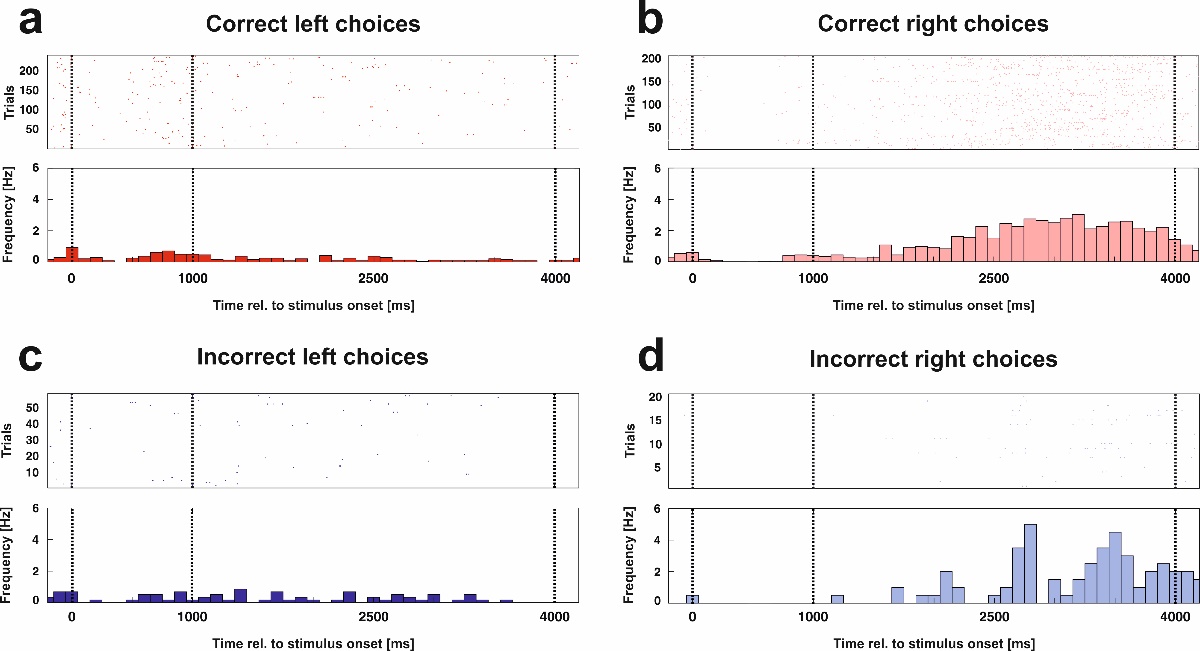


Supplementary Figure 7. Responses of another example neuron for all left and right choices across all experimental stimuli.

a The raster plot and PSTH for correct choices to the left response key are shown. b The corresponding responses to the right response key are depicted. c The raster plot and PSTH for incorrect choices to the left response key are shown. d The corresponding responses to the right response key are depicted. Again, this neuron’s spiking activity corresponded to the choice of the animal regardless whether it turned out to be correct or incorrect.


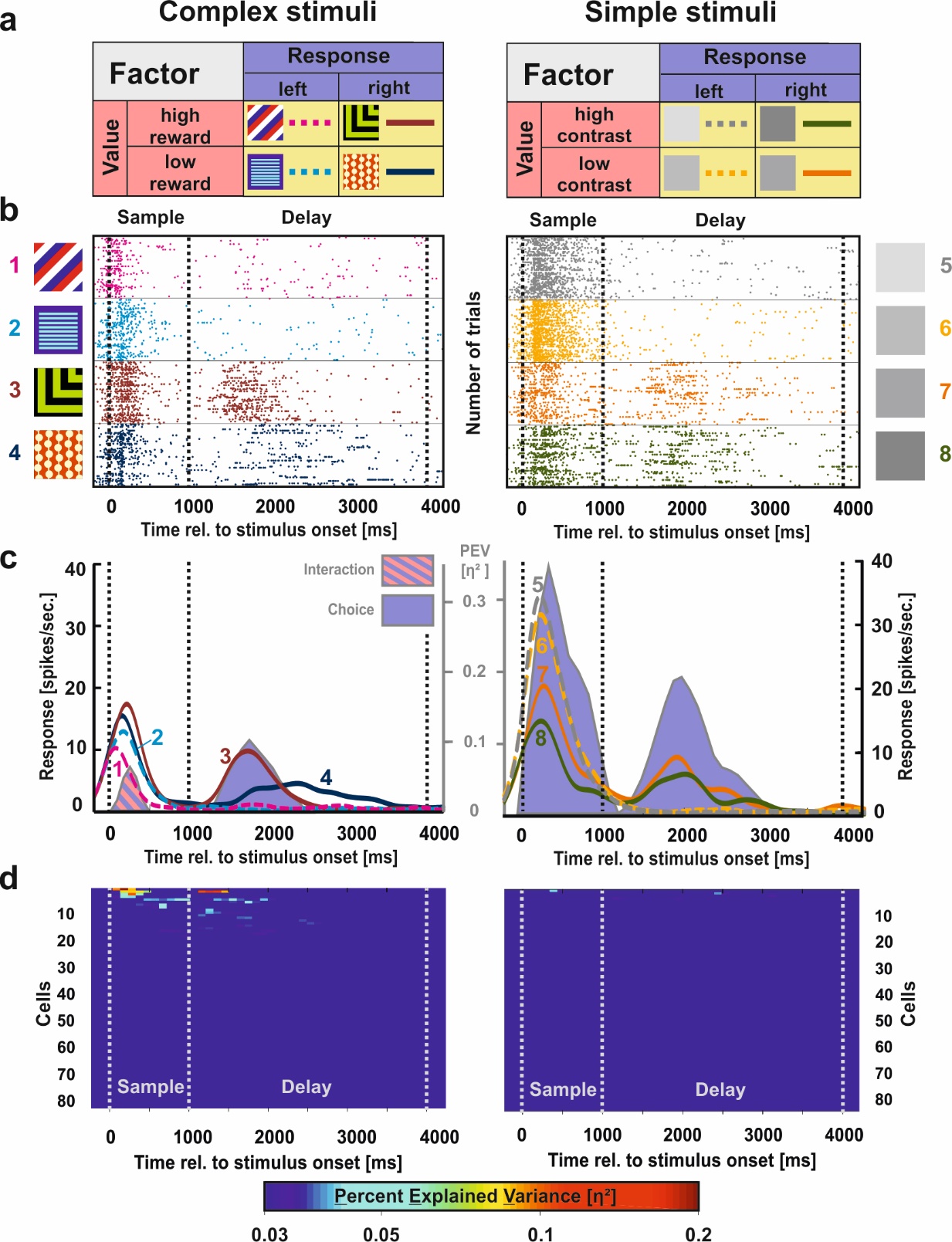


Supplementary Figure 8. Example neuron and population response for interaction activity in the NCL.

a) The rationale of the ANOVA as described in Figure 3. b) Raster plot of an example neuron illustrating interaction activity. For complex stimuli, this neuron increased its firing rate for one high reward stimulus whereas it decreased the firing rate for the other high reward stimulus compared to both low reward stimuli resulting in a significant interaction effect during the sample phase (left). For simple stimuli, choice-related activity was present already during the sample phase (right). During the delay, this neuron demonstrated a choice code for complex and simple stimuli alike. c) Spike density function of the example neuron shown in b. The PEV by the relevant coding type is presented behind the SDF as shaded area in the respective color of the activity pattern (stimulus = yellow, value = red, choice = blue; cf. panel a). Corresponding values are scaled on the secondary axis depicted in gray. d) Population response for interaction activity of all individual neurons for trials in which complex (left panel) and simple stimuli (right panel) were presented.


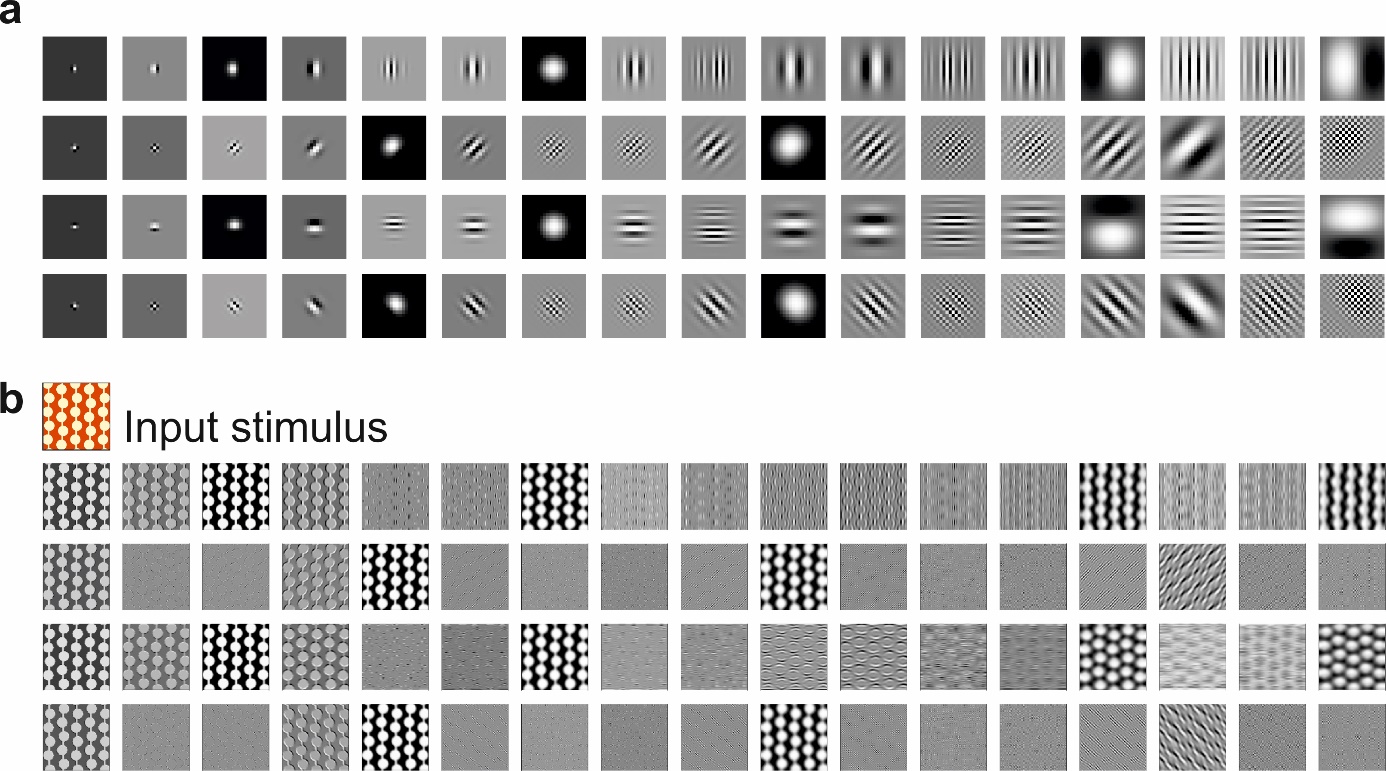


Supplementary Figure 9. Gabor filters used to estimate of the stimulus-specific spatial visual information of the stimulus set.

a) 68 Gabor filters used to estimate the stimulus-specific visual information of the stimulus set. b) Sample outputs after convolution of one stimulus with each Gabor filter.

***
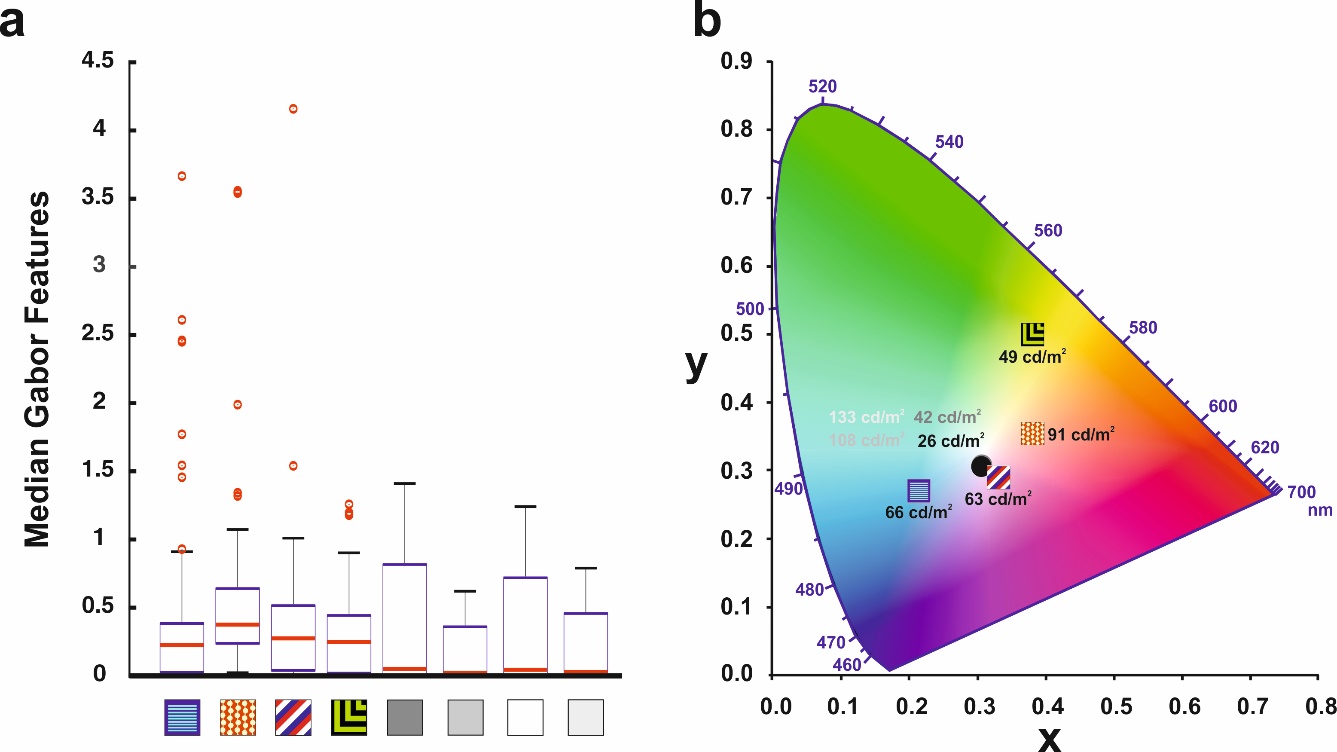
***

Supplementary Figure 10. Images’ visual information both in the spatial and color dimension.

a) The output of the 68 Gabor filters for all stimuli. The complex stimuli are characterized by spatial image features reflected by a large number of responsive Gabor filters resulting in a high median activity. The simple stimuli are void of any spatial features resulting in a marginal median Gabor filter activity. Whiskers represent Q1 − 1.5 * interquartile range (IQR) and Q3 + 1.5 * IQR. b) Representation of the stimulus set in the CIE 1931 color space chromaticity diagram. The complex stimuli are arranged within the color space and deviate from the white point of the figure. In contrast, all the simple stimuli are collapsed in the white point of the color space. Thus, besides their luminance difference, no additional color information is contained in these stimuli.

Supplementary Table 1. Number of sessions collected from each individual animal across the three behavioral experiments as well as the recording experiment.

| Animal ID | Experiment 1 | Experiment 2 | Experiment 3 | Recordings |
| --- | --- | --- | --- | --- |
| 009 | 2 | - | 1 | - |
| 046 | 9 | - | 1 | - |
| 068 | 2 | - | - | - |
| 111 | - | 3 | - | - |
| 173 | - | - | 2 | - |
| 182 | - | - | - | 5 |
| 204 | - | - | 8 | - |
| 215 | - | - | 16 | - |
| 335 | - | - | 32 | - |
| 356 | - | - | 5 | - |
| 381 | - | - | 5 | - |
| 459 | - | - | 13 | - |
| 467 | - | - | 5 | - |
| 522 | 5 | 8 | 3 | - |
| 526 | 16 | - | - | - |
| 640 | 9 | 5 | 3 | - |
| 666 | - | - | - | 5 |
| 675 | - | 2 | 2 | - |
| 693 | - | - | 1 | - |
| 721 | 9 | 9 | 3 | - |
| 754 | 7 | - | - | - |
| 804 | - | 4 | 3 | - |
| 850 | - | - | - | 24 |
| 851 | - | 2 | - | - |
| 856 | 14 | 6 | - | - |
| 859 | 13 | - | - | - |
| 916 | 3 | - | 1 | - |
| Pigeons Total: 27 | Pigeons Exp. 1: 11 | Pigeons Exp. 2: 8 | Pigeons Exp. 3: 17 | Pigeons Rec.: 3 |
| Sessions | Sessions: 89 | Sessions: 39 | Sessions: 104 | Sessions: 34 |
| Ø sessions/pigeon | 8,1 | 4,9 | 6,1 | 10,7 |

Supplementary Table 2. Behavioral performance in experiment 1 for each stimulus pair during either the “no delay” or “delay” conditions.

| Stimulus | Delay | Mean Performance (SD) | Δ Delay – No Delay |
| --- | --- | --- | --- |
| Complex pair 1 | no | 93.5% (5.5%) | 1.2% |
| Complex pair 1 | yes | 92.3% (4.3%) |  |
| Complex pair 2 | no | 93.6% (4.3%) | 3.1% |
| Complex pair 2 | yes | 90.5% (6.2%) |  |
| Simple high contrast | no | 94.4% (4.0%) | 8.7% |
| Simple high contrast | yes | 85.7% (5.6%) |  |
| Simple low contrast | no | 90.4% (5.8%) | 9.2% |
| Simple low contrast | yes | 81.2% (7.4%) |  |

Supplementary Table 3. Behavioral performance in experiment 2 for each stimulus pair during either the “no delay” or “delay” conditions.

| Stimulus | Delay | Mean Performance (SD) | Δ Delay – No Delay |
| --- | --- | --- | --- |
| Complex pair 1 | no | 96.0% (4.0%) | 1.9% |
| Complex pair 1 | yes | 94.1% (3.7%) |  |
| Complex pair 2 | no | 93.1% (4.9%) | 1.2% |
| Complex pair 2 | yes | 91.9% (6.2%) |  |
| Simple high contrast | no | 95.3% (3.5%) | 8.4% |
| Simple high contrast | yes | 86.9% (11.0%) |  |
| Simple low contrast | no | 93.0% (4.3%) | 11.3% |
| Simple low contrast | yes | 81.7% (11.0%) |  |

Supplementary Table 4. Behavioral performance in experiment 3 for each stimulus pair during either the “no delay” or “delay” conditions.

| Stimulus | Delay | Mean Performance (SD) | Δ Delay – No Delay |
| --- | --- | --- | --- |
| Complex pair (high reward) | no | 96.1% (3.2%) | 2.3% |
| Complex pair (high reward) | yes | 93.8% (4.9%) |  |
| Complex pair (low reward) | no | 88.8% (6.7%) | -0.8% |
| Complex pair (low reward) | yes | 89.6% (6.5%) |  |
| Simple high contrast | no | 94.9% (3.6%) | 6.7% |
| Simple high contrast | yes | 88.2% (5.9%) |  |
| Simple low contrast | no | 87.6% (5.0%) | 9.4% |
| Simple low contrast | yes | 78.2% (7.1%) |  |

**Supplementary Table 5**. Distribution of active cells per category across the sample and delay phase for the simulated data in both the complex and simple stimuli. Note that the indicated value represents an average across 200 simulations.


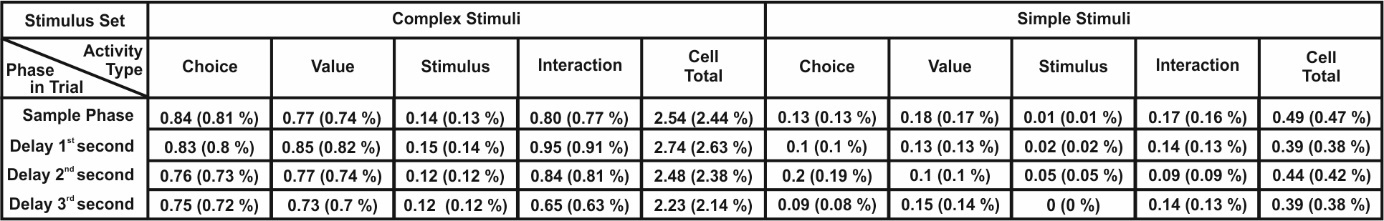


**Supplementary References**

[1] Güntürkün, O., Verhoye, M., De Groof, G. & van der Linden, A. A 3-dimensional digital atlas of the ascending sensory and the descending motor systems in the pigeon brain. *Brain Struct. Funct.* **218**, 269–281 (2013).

[2] Karten, H. J. & Hodos, W. A stereotaxic atlas of the brain of the pigeon (*Columba livia*). Baltimore: Johns Hopkins Press (1967).

[3] Herold, C., Palomero-Gallagher, N., Güntürkün, O. & Zilles, K. Serotonin 5-HT1A receptor binding sites in the brain of the pigeon (*Columba livia*). *Neuroscience* **200**, 1-12 (2012).
