# Supplementary material for: Working memory performance is tied to stimulus complexity
Source: Commun Biol. 2023 Nov 3;6:1119. doi: 10.1038/s42003-023-05486-7 (PMC10624839; doi:10.1038/s42003-023-05486-7)
Supplement: Supplementary file 3 — Description of additional supplementary files [file 42003_2023_5486_MOESM3_ESM.docx]

Description of Additional Supplementary Files

**File name:** Supplementary Data 1

**Description**: The source data behind the Supplementary Figures1, 2, 3, 5, 6, 7, 8 & 10
