# Supplementary material for: Working memory performance is tied to stimulus complexity
Source: Commun Biol. 2023 Nov 3;6:1119. doi: 10.1038/s42003-023-05486-7 (PMC10624839; doi:10.1038/s42003-023-05486-7)
Supplement: Supplementary file 5 — Reporting Summary [file 42003_2023_5486_MOESM5_ESM.pdf]

nature portfolio

Corresponding author(s): Roland Pusch

Last updated by author(s): 18.10.23

Reporting Summary

Nature Portfolio wishes to improve the reproducibility of the work that we publish. This form provides structure for consistency and transparency in reporting. For further information on Nature Portfolio policies, see our Editorial Policies and the Editorial Policy Checklist.

Statistics

For all statistical analyses, confirm that the following items are present in the figure legend, table legend, main text, or Methods section.

n/a	Confirmed	
☐	☒	The exact sample size (*n*) for each experimental group/condition, given as a discrete number and unit of measurement
☐	☒	A statement on whether measurements were taken from distinct samples or whether the same sample was measured repeatedly
☐	☒	The statistical test(s) used AND whether they are one- or two-sided<br>*Only common tests should be described solely by name; describe more complex techniques in the Methods section.*
☐	☒	A description of all covariates tested
☐	☒	A description of any assumptions or corrections, such as tests of normality and adjustment for multiple comparisons
☐	☒	A full description of the statistical parameters including central tendency (e.g. means) or other basic estimates (e.g. regression coefficient) AND variation (e.g. standard deviation) or associated estimates of uncertainty (e.g. confidence intervals)
☐	☒	For null hypothesis testing, the test statistic (e.g. *F*, *t*, *r*) with confidence intervals, effect sizes, degrees of freedom and *P* value noted<br>*Give P values as exact values whenever suitable.*
☒	☐	For Bayesian analysis, information on the choice of priors and Markov chain Monte Carlo settings
☒	☐	For hierarchical and complex designs, identification of the appropriate level for tests and full reporting of outcomes
☐	☒	Estimates of effect sizes (e.g. Cohen's *d*, Pearson's *r*), indicating how they were calculated

Our web collection on statistics for biologists contains articles on many of the points above.

Software and code

Policy information about availability of computer code

Data collection	MATLAB (Biopsychology Toolbox, MLIB Toolbox), Spike 2, Alpha Lab SnR
Data analysis	MATLAB2022b, R 4.1.0; 2018a

For manuscripts utilizing custom algorithms or software that are central to the research but not yet described in published literature, software must be made available to editors and reviewers. We strongly encourage code deposition in a community repository (e.g. GitHub). See the Nature Portfolio guidelines for submitting code & software for further information.

Data

Policy information about availability of data

All manuscripts must include a data availability statement. This statement should provide the following information, where applicable:

- Accession codes, unique identifiers, or web links for publicly available datasets
- A description of any restrictions on data availability
- For clinical datasets or third party data, please ensure that the statement adheres to our policy

Data will be made available through contacting the lead author (Roland Pusch).

Research involving human participants, their data, or biological material

Policy information about studies with human participants or human data. See also policy information about sex, gender (identity/presentation), and sexual orientation and race, ethnicity and racism.

Reporting on sex and gender	N/A
Reporting on race, ethnicity, or other socially relevant groupings	N/A
Population characteristics	N/A
Recruitment	N/A
Ethics oversight	N/A

Note that full information on the approval of the study protocol must also be provided in the manuscript.

Field-specific reporting

Please select the one below that is the best fit for your research. If you are not sure, read the appropriate sections before making your selection.

☒ Life sciences ☐ Behavioural & social sciences ☐ Ecological, evolutionary & environmental sciences

For a reference copy of the document with all sections, see nature.com/documents/nr-reporting-summary-flat.pdf

Life sciences study design

All studies must disclose on these points even when the disclosure is negative.

Sample size	No a priori sample size was calculated. The sample sizes in the present manuscript are high for both the behavioral experiments as well for neuron numbers in the physiological recordings. The observed effects are generally large and posthoc power calculations were consistently above 90%. Findings were highly consistent across sub-experiments.
Data exclusions	To enter further behavioral analysis, all sessions had to meet the following criteria: At least 70% correct choices had to be reached to ensure that choices reflected learned behavior. Sessions that did not meet this criterion were excluded.
Replication	Findings from Experiment 1 were conceptually reproduced in different animals across Experiment 2, 3 and the physiological recording experiments. Thus, the results were highly robust.
Randomization	All investigated effects are investigated within subject as all stimuli were presented to every animal. Stimulus presentation was randomized across each session.
Blinding	Experimenters were not blinded to the analysis. This is however irrelevant as the experiments constitute operant conditioning paradigms where correct or incorrect trials are decided based on computer-registered results and are calculated using objective code. Blinding would not influence the results.

Reporting for specific materials, systems and methods

We require information from authors about some types of materials, experimental systems and methods used in many studies. Here, indicate whether each material, system or method listed is relevant to your study. If you are not sure if a list item applies to your research, read the appropriate section before selecting a response.

Materials & experimental systems

n/a	Involved in the study	
☒	☐	Antibodies
☒	☐	Eukaryotic cell lines
☒	☐	Palaeontology and archaeology
☐	☒	Animals and other organisms
☒	☐	Clinical data
☒	☐	Dual use research of concern
☒	☐	Plants

Methods

n/a	Involved in the study	
☒	☐	ChIP-seq
☒	☐	Flow cytometry
☒	☐	MRI-based neuroimaging

Animals and other research organisms

Policy information about studies involving animals; ARRIVE guidelines recommended for reporting animal research, and Sex and Gender in Research

Laboratory animals	Pigeons (Columba livia) were used in the present study. The animals were of unknown sex. The pigeons were between 1 and 6 years of age.
Wild animals	N/A
Reporting on sex	Sex was not considered as the sex of the animals was unknown.
Field-collected samples	N/A
Ethics oversight	Subjects were treated in accordance with the German guidelines for the care and use of animals in science and all experimental procedures were approved by a national ethics committee of the State of North Rhine-Westphalia, Germany (LANUV) and were in agreement with the Directive 2010/63/EU of the European Parliament and of the Council of 22 September 2010 concerning the care and use of animals for experimental purposes. We have complied with all relevant ethical regulations for animal testing.

Note that full information on the approval of the study protocol must also be provided in the manuscript.
